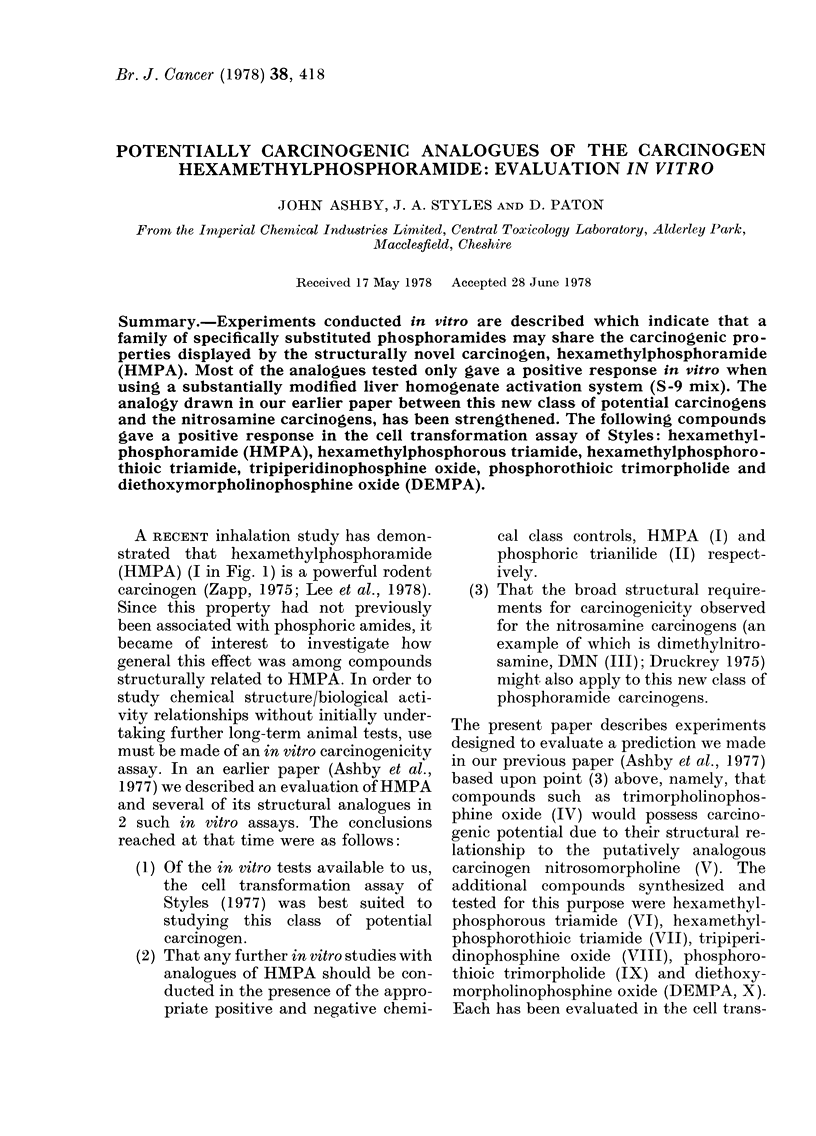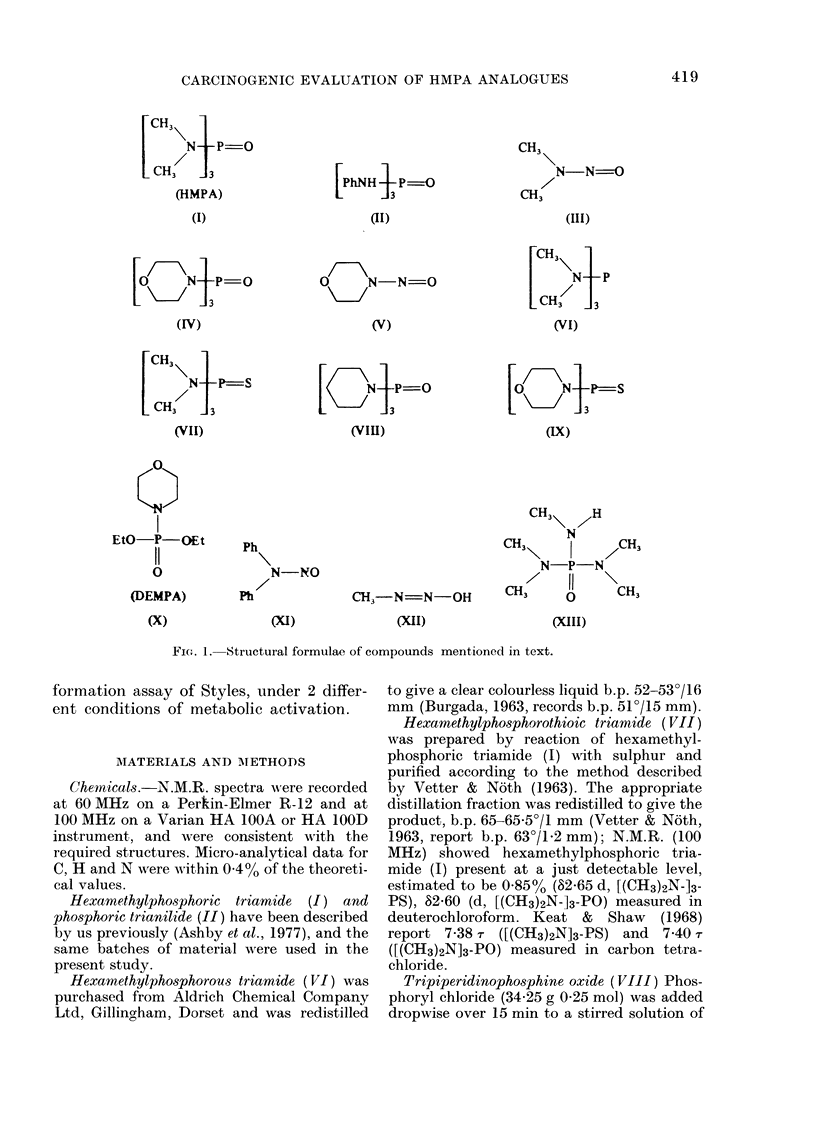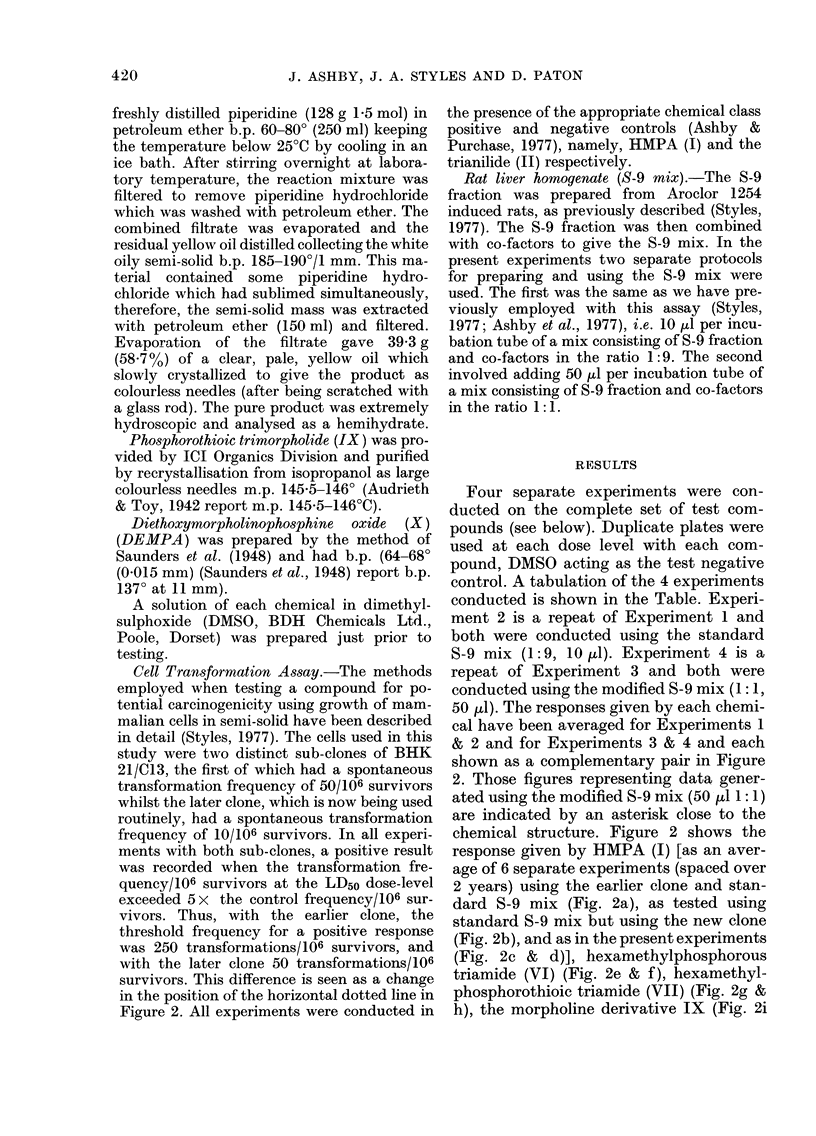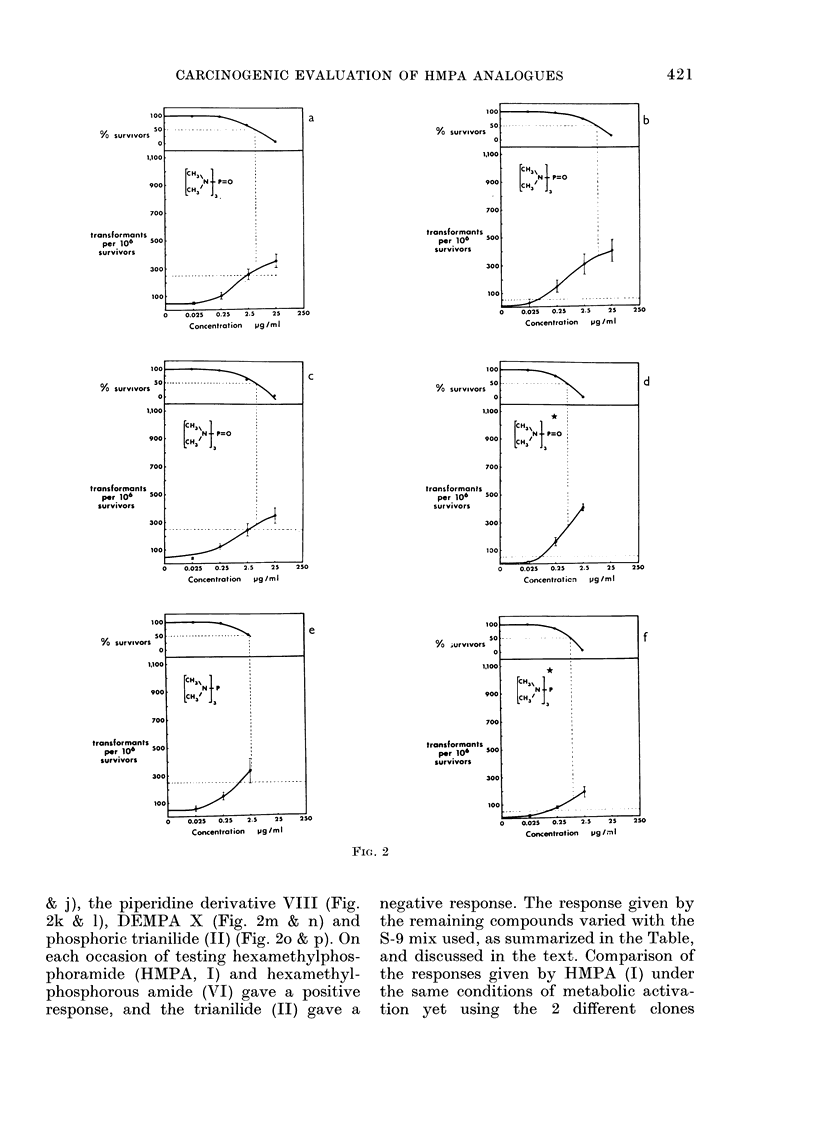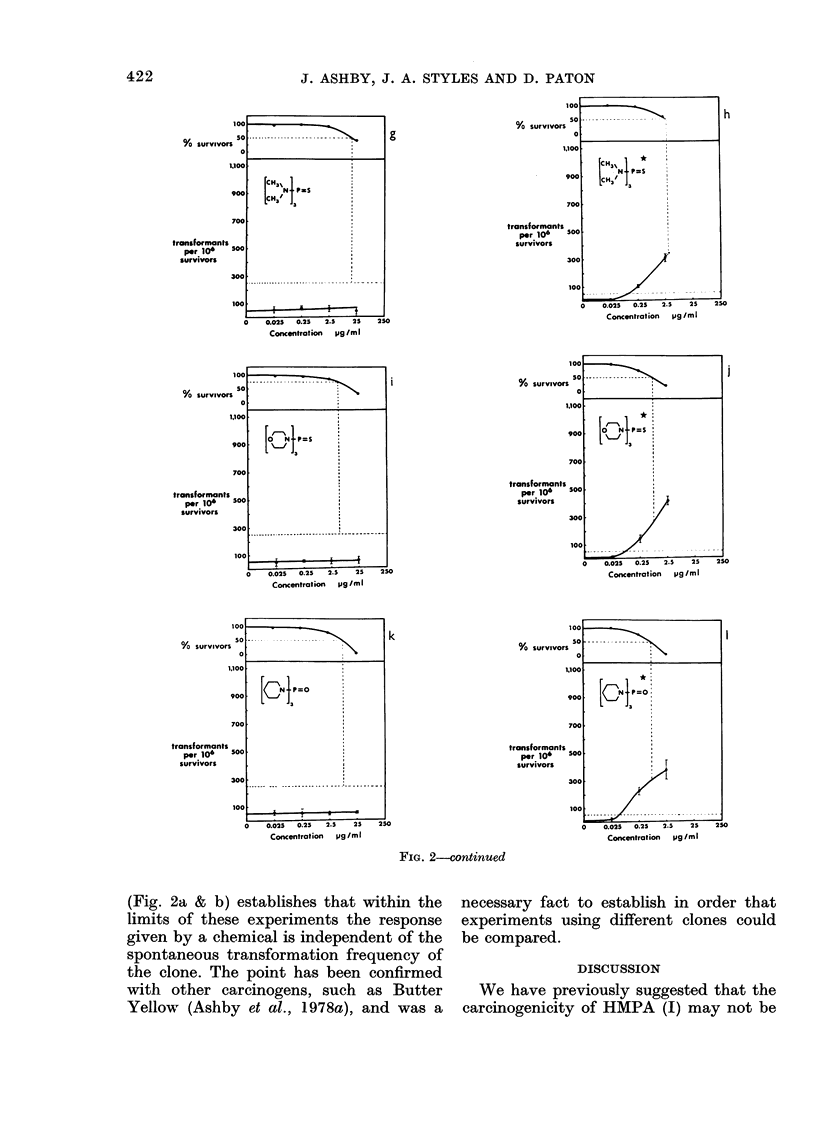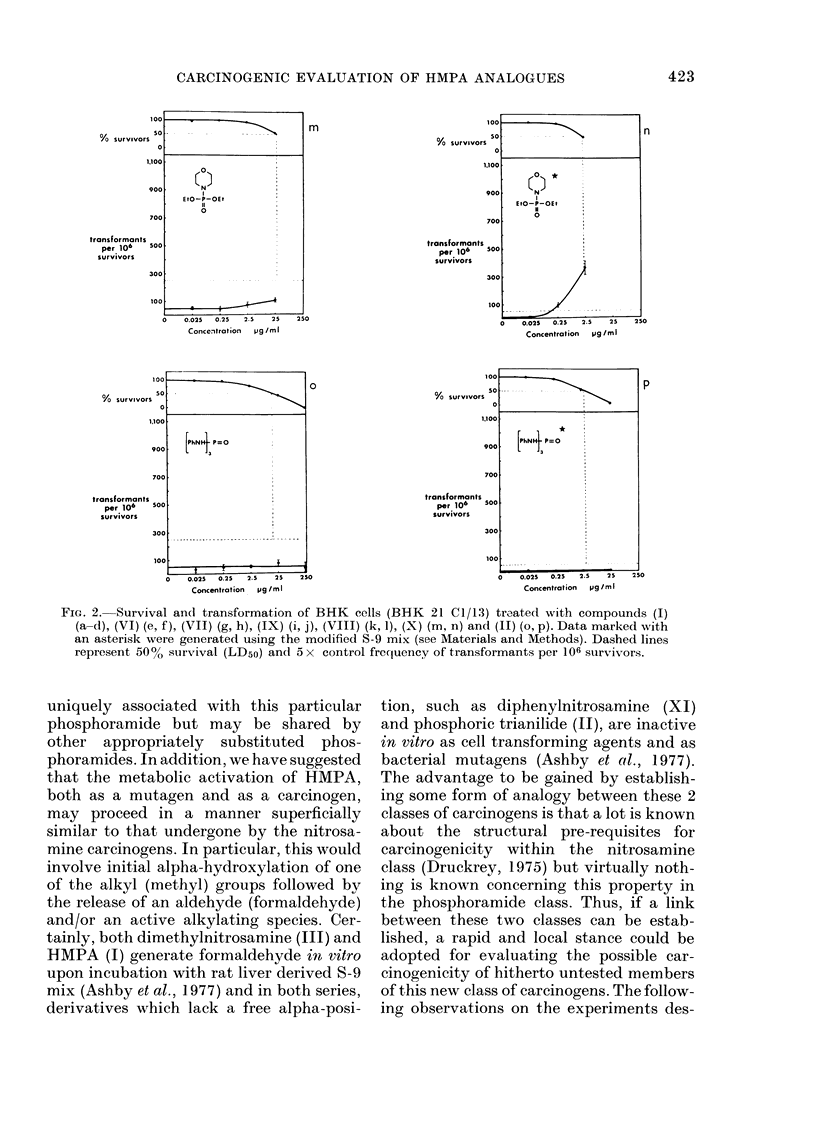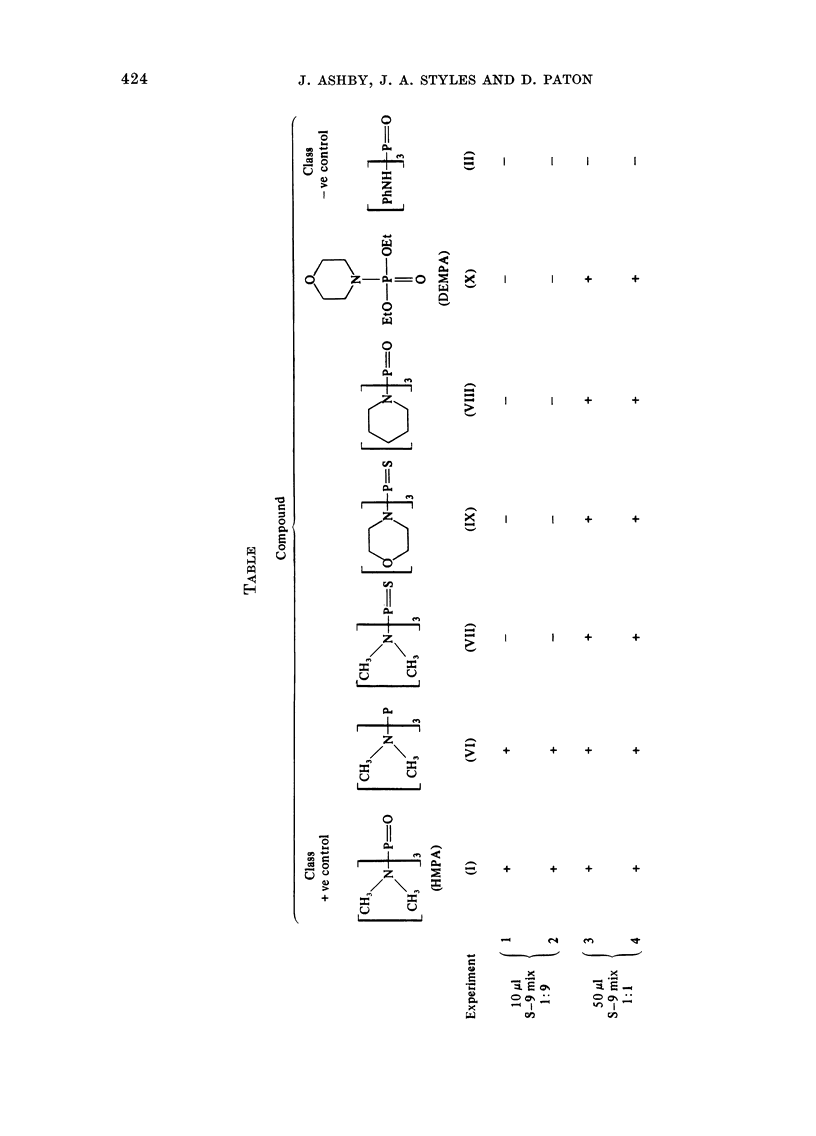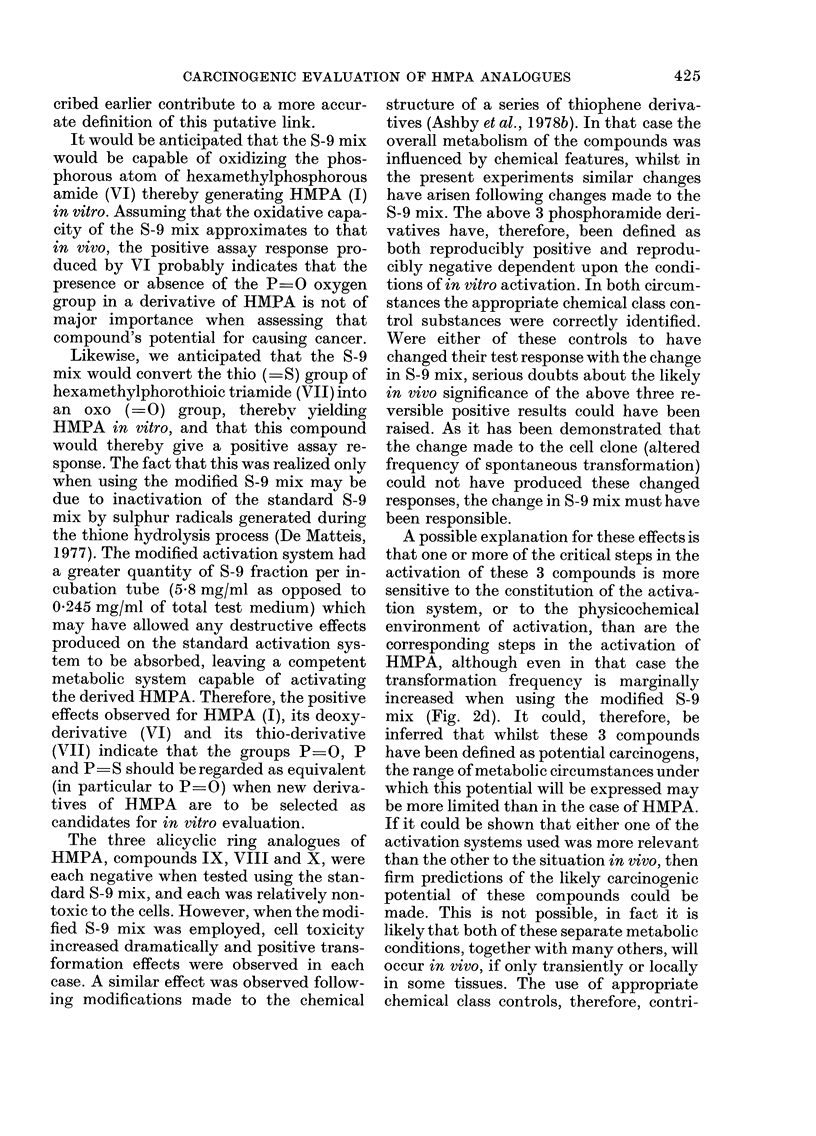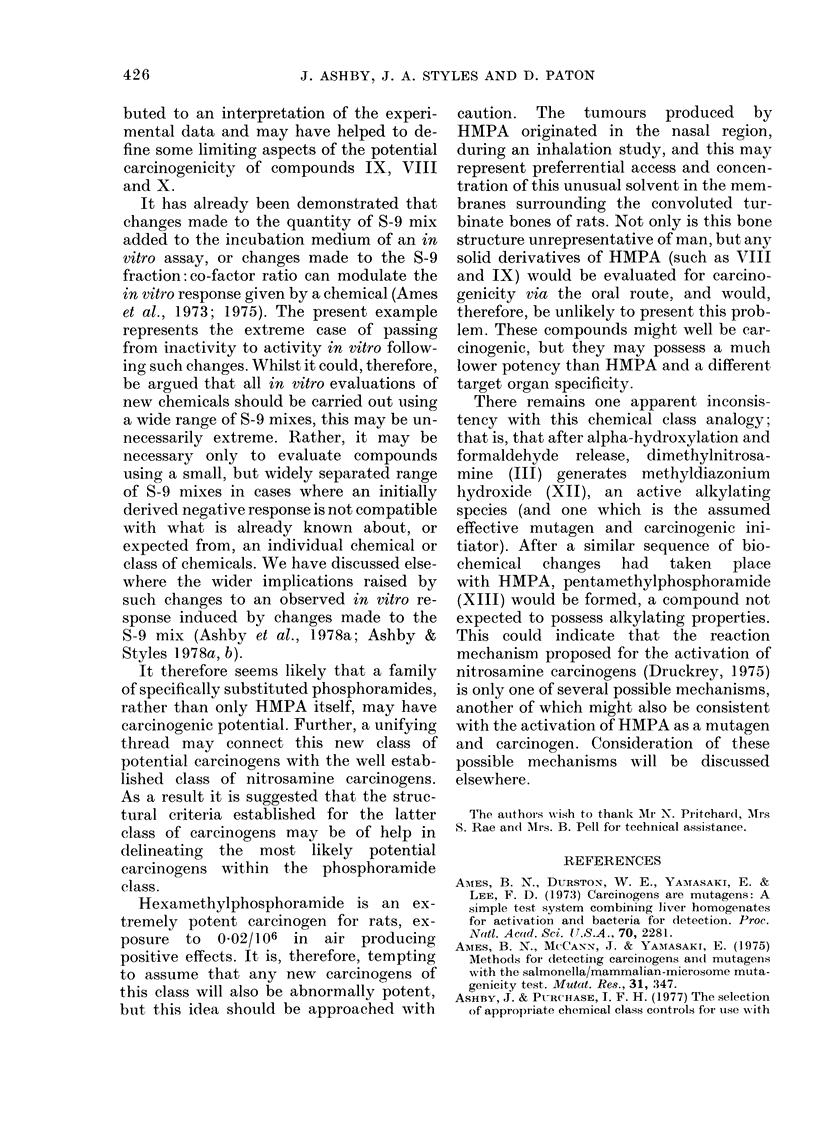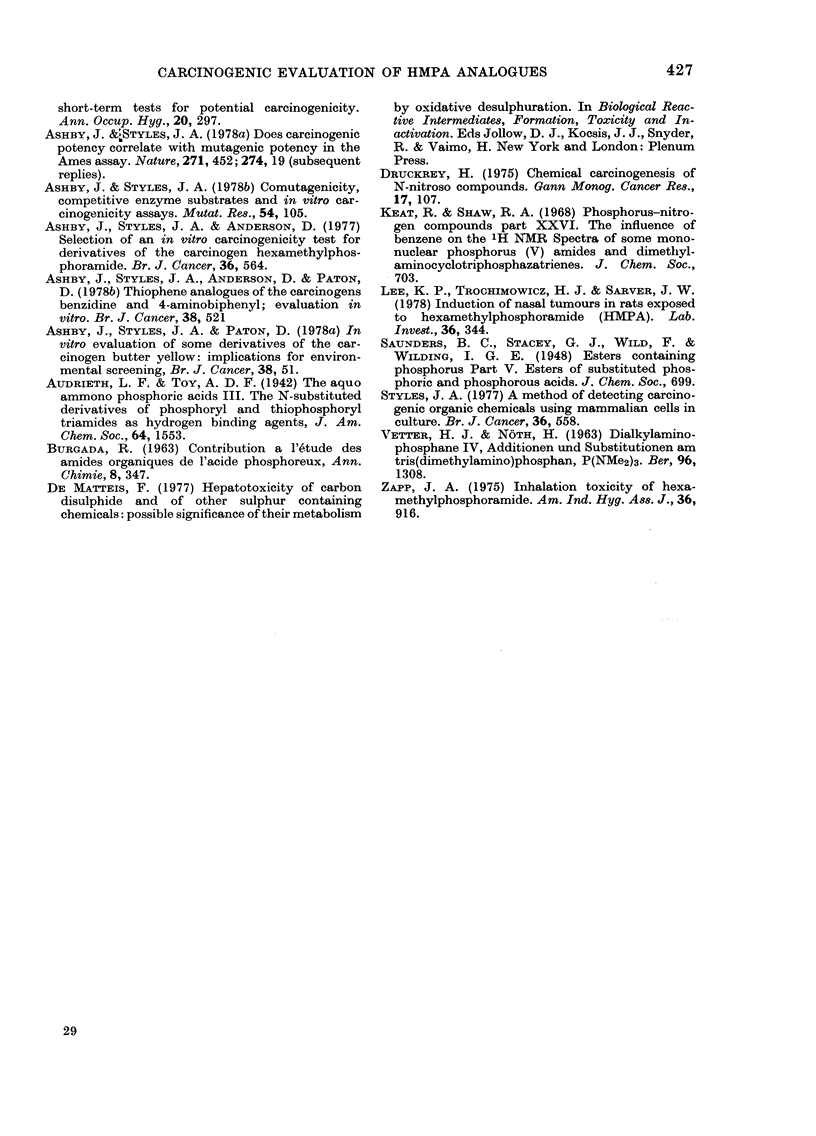# Potentially carcinogenic analogues of the carcinogen hexamethylphosphoramide: evaluation in vitro.

**DOI:** 10.1038/bjc.1978.223

**Published:** 1978-09

**Authors:** J. Ashby, J. A. Styles, D. Paton

## Abstract

Experiments conducted in vitro are described which indicate that a family of specifically substituted phosphoramides may share the carcinogenic properties displayed by the structurally novel carcinogen, hexamethylphosphoramide (HMPA). Most of the analogues tested only gave a positive response in vitro when using a substantially modified liver homogenate activation system (S-9 mix). The analogy drawn in our earlier paper between this new class of potential carcinogens and the nitrosamine carcinogens, has been strengthened. The following compounds gave a positive response in the cell transformation assay of Styles: hexamethylphosphoramide (HMPA), hexamethylphosphorous triamide, hexamethylphosphorothioic triamide, tripiperidinophosphine oxide, phosphorothioic trimorpholide and diethoxymorpholinophosphine oxide (DEMPA).


*Br. J. Cancer* (1978) **38**, 418

### POTENTIALLY CARCINOGENIC ANALOGUES OF THE CARCINOGEN HEXAMETHYLPHOSPHORAMIDE: EVALUATION *IN VITRO*

JOHN ASHBY, J. A. STYLES AND D. PATON

*From the Imperial Chemical Industries Limited, Central Toxicology Laboratory, Alderley Park, Macclesfield, Cheshire*

Received 17 May 1978 Accepted 28 June 1978

**Summary.—Experiments conducted *in vitro* are described which indicate that a family of specifically substituted phosphoramides may share the carcinogenic properties displayed by the structurally novel carcinogen, hexamethylphosphoramide (HMPA). Most of the analogues tested only gave a positive response *in vitro* when using a substantially modified liver homogenate activation system (S-9 mix). The analogy drawn in our earlier paper between this new class of potential carcinogens and the nitrosamine carcinogens, has been strengthened. The following compounds gave a positive response in the cell transformation assay of Styles: hexamethylphosphoramide (HMPA), hexamethylphosphorous triamide, hexamethylphosphorothioic triamide, tripiperidinophosphine oxide, phosphorothioic trimorpholide and diethoxymorpholinophosphine oxide (DEMPA).**

A RECENT inhalation study has demonstrated that hexamethylphosphoramide (HMPA) (I in Fig. 1) is a powerful rodent carcinogen (Zapp, 1975; Lee *et al.*, 1978). Since this property had not previously been associated with phosphoric amides, it became of interest to investigate how general this effect was among compounds structurally related to HMPA. In order to study chemical structure/biological activity relationships without initially undertaking further long-term animal tests, use must be made of an *in vitro* carcinogenicity assay. In an earlier paper (Ashby *et al.*, 1977) we described an evaluation of HMPA and several of its structural analogues in 2 such *in vitro* assays. The conclusions reached at that time were as follows:

(1) Of the *in vitro* tests available to us, the cell transformation assay of Styles (1977) was best suited to studying this class of potential carcinogen.
(2) That any further *in vitro* studies with analogues of HMPA should be conducted in the presence of the appropriate positive and negative chemical class controls, HMPA (I) and phosphoric trianilide (II) respectively.
(3) That the broad structural requirements for carcinogenicity observed for the nitrosamine carcinogens (an example of which is dimethylnitrosamine, DMN (III); Druckrey 1975) might also apply to this new class of phosphoramide carcinogens.

The present paper describes experiments designed to evaluate a prediction we made in our previous paper (Ashby *et al.*, 1977) based upon point (3) above, namely, that compounds such as trimorpholinophosphine oxide (IV) would possess carcinogenic potential due to their structural relationship to the putatively analogous carcinogen nitrosomorpholine (V). The additional compounds synthesized and tested for this purpose were hexamethylphosphorous triamide (VI), hexamethylphosphorothioic triamide (VII), tripiperidinophosphine oxide (VIII), phosphorothioic trimorpholide (IX) and diethoxymorpholinophosphine oxide (DEMPA, X). Each has been evaluated in the cell trans-

FIG. 1.—Structural formulae of compounds mentioned in text.

formation assay of Styles, under 2 different conditions of metabolic activation.

### MATERIALS AND METHODS

*Chemicals.*—N.M.R. spectra were recorded at 60 MHz on a Perkin-Elmer R-12 and at 100 MHz on a Varian HA 100A or HA 100D instrument, and were consistent with the required structures. Micro-analytical data for C, H and N were within 0·4% of the theoretical values.

*Hexamethylphosphoric triamide (I) and phosphoric trianilide (II)* have been described by us previously (Ashby *et al.*, 1977), and the same batches of material were used in the present study.

*Hexamethylphosphorous triamide (VI)* was purchased from Aldrich Chemical Company Ltd, Gillingham, Dorset and was redistilled to give a clear colourless liquid b.p. 52–53°/16 mm (Burgada, 1963, records b.p. 51°/15 mm).

*Hexamethylphosphorothioic triamide (VII)* was prepared by reaction of hexamethylphosphoric triamide (I) with sulphur and purified according to the method described by Vetter & Nöth (1963). The appropriate distillation fraction was redistilled to give the product, b.p. 65–65·5°/1 mm (Vetter & Nöth, 1963, report b.p. 63°/1·2 mm); N.M.R. (100 MHz) showed hexamethylphosphoric triamide (I) present at a just detectable level, estimated to be 0·85% (δ2·65 d, $[(CH_3)_2N\text{-}]_3\text{-}PS$), δ2·60 (d, $[(CH_3)_2N\text{-}]_3\text{-}PO$) measured in deuterochloroform. Keat & Shaw (1968) report 7·38 τ ($[(CH_3)_2N]_3\text{-}PS$) and 7·40 τ ($[(CH_3)_2N]_3\text{-}PO$) measured in carbon tetrachloride.

*Tripiperidinophosphine oxide (VIII)* Phosphoryl chloride (34·25 g 0·25 mol) was added dropwise over 15 min to a stirred solution of freshly distilled piperidine (128 g 1·5 mol) in petroleum ether b.p. 60–80° (250 ml) keeping the temperature below 25°C by cooling in an ice bath. After stirring overnight at laboratory temperature, the reaction mixture was filtered to remove piperidine hydrochloride which was washed with petroleum ether. The combined filtrate was evaporated and the residual yellow oil distilled collecting the white oily semi-solid b.p. 185–190°/1 mm. This material contained some piperidine hydrochloride which had sublimed simultaneously, therefore, the semi-solid mass was extracted with petroleum ether (150 ml) and filtered. Evaporation of the filtrate gave 39·3 g (58·7%) of a clear, pale, yellow oil which slowly crystallized to give the product as colourless needles (after being scratched with a glass rod). The pure product was extremely hydroscopic and analysed as a hemihydrate.

*Phosphorothioic trimorpholide (IX)* was provided by ICI Organics Division and purified by recrystallisation from isopropanol as large colourless needles m.p. 145·5–146° (Audrieth & Toy, 1942 report m.p. 145·5–146°C).

*Diethoxymorpholinophosphine oxide (X) (DEMPA)* was prepared by the method of Saunders *et al.* (1948) and had b.p. (64–68° (0·015 mm) (Saunders *et al.*, 1948) report b.p. 137° at 11 mm).

A solution of each chemical in dimethylsulphoxide (DMSO, BDH Chemicals Ltd., Poole, Dorset) was prepared just prior to testing.

*Cell Transformation Assay.*—The methods employed when testing a compound for potential carcinogenicity using growth of mammalian cells in semi-solid have been described in detail (Styles, 1977). The cells used in this study were two distinct sub-clones of BHK 21/C13, the first of which had a spontaneous transformation frequency of $50/10^6$ survivors whilst the later clone, which is now being used routinely, had a spontaneous transformation frequency of $10/10^6$ survivors. In all experiments with both sub-clones, a positive result was recorded when the transformation frequency/$10^6$ survivors at the $LD_{50}$ dose-level exceeded 5× the control frequency/$10^6$ survivors. Thus, with the earlier clone, the threshold frequency for a positive response was 250 transformations/$10^6$ survivors, and with the later clone 50 transformations/$10^6$ survivors. This difference is seen as a change in the position of the horizontal dotted line in Figure 2. All experiments were conducted in the presence of the appropriate chemical class positive and negative controls (Ashby & Purchase, 1977), namely, HMPA (I) and the trianilide (II) respectively.

*Rat liver homogenate (S-9 mix).*—The S-9 fraction was prepared from Aroclor 1254 induced rats, as previously described (Styles, 1977). The S-9 fraction was then combined with co-factors to give the S-9 mix. In the present experiments two separate protocols for preparing and using the S-9 mix were used. The first was the same as we have previously employed with this assay (Styles, 1977; Ashby *et al.*, 1977), *i.e.* 10 μl per incubation tube of a mix consisting of S-9 fraction and co-factors in the ratio 1:9. The second involved adding 50 μl per incubation tube of a mix consisting of S-9 fraction and co-factors in the ratio 1:1.

### RESULTS

Four separate experiments were conducted on the complete set of test compounds (see below). Duplicate plates were used at each dose level with each compound, DMSO acting as the test negative control. A tabulation of the 4 experiments conducted is shown in the Table. Experiment 2 is a repeat of Experiment 1 and both were conducted using the standard S-9 mix (1:9, 10 μl). Experiment 4 is a repeat of Experiment 3 and both were conducted using the modified S-9 mix (1:1, 50 μl). The responses given by each chemical have been averaged for Experiments 1 & 2 and for Experiments 3 & 4 and each shown as a complementary pair in Figure 2. Those figures representing data generated using the modified S-9 mix (50 μl 1:1) are indicated by an asterisk close to the chemical structure. Figure 2 shows the response given by HMPA (I) [as an average of 6 separate experiments (spaced over 2 years) using the earlier clone and standard S-9 mix (Fig. 2a), as tested using standard S-9 mix but using the new clone (Fig. 2b), and as in the present experiments (Fig. 2c & d)], hexamethylphosphorous triamide (VI) (Fig. 2e & f), hexamethylphosphorothioic triamide (VII) (Fig. 2g & h), the morpholine derivative IX (Fig. 2i

FIG. 2

& j), the piperidine derivative VIII (Fig. 2k & l), DEMPA X (Fig. 2m & n) and phosphoric trianilide (II) (Fig. 2o & p). On each occasion of testing hexamethylphosphoramide (HMPA, I) and hexamethylphosphorous amide (VI) gave a positive response, and the trianilide (II) gave a negative response. The response given by the remaining compounds varied with the S-9 mix used, as summarized in the Table, and discussed in the text. Comparison of the responses given by HMPA (I) under the same conditions of metabolic activation yet using the 2 different clones

FIG. 2—*continued*

(Fig. 2a & b) establishes that within the limits of these experiments the response given by a chemical is independent of the spontaneous transformation frequency of the clone. The point has been confirmed with other carcinogens, such as Butter Yellow (Ashby *et al.*, 1978*a*), and was a necessary fact to establish in order that experiments using different clones could be compared.

### DISCUSSION

We have previously suggested that the carcinogenicity of HMPA (I) may not be

FIG. 2.—Survival and transformation of BHK cells (BHK 21 Cl/13) treated with compounds (I) (a–d), (VI) (e, f), (VII) (g, h), (IX) (i, j), (VIII) (k, l), (X) (m, n) and (II) (o, p). Data marked with an asterisk were generated using the modified S-9 mix (see Materials and Methods). Dashed lines represent 50% survival ($LD_{50}$) and 5× control frequency of transformants per $10^6$ survivors.

uniquely associated with this particular phosphoramide but may be shared by other appropriately substituted phosphoramides. In addition, we have suggested that the metabolic activation of HMPA, both as a mutagen and as a carcinogen, may proceed in a manner superficially similar to that undergone by the nitrosamine carcinogens. In particular, this would involve initial alpha-hydroxylation of one of the alkyl (methyl) groups followed by the release of an aldehyde (formaldehyde) and/or an active alkylating species. Certainly, both dimethylnitrosamine (III) and HMPA (I) generate formaldehyde *in vitro* upon incubation with rat liver derived S-9 mix (Ashby *et al.*, 1977) and in both series, derivatives which lack a free alpha-position, such as diphenylnitrosamine (XI) and phosphoric trianilide (II), are inactive *in vitro* as cell transforming agents and as bacterial mutagens (Ashby *et al.*, 1977). The advantage to be gained by establishing some form of analogy between these 2 classes of carcinogens is that a lot is known about the structural pre-requisites for carcinogenicity within the nitrosamine class (Druckrey, 1975) but virtually nothing is known concerning this property in the phosphoramide class. Thus, if a link between these two classes can be established, a rapid and local stance could be adopted for evaluating the possible carcinogenicity of hitherto untested members of this new class of carcinogens. The following observations on the experiments des-

TABLE

| | | Compound | | | | | |
|---|---|---|---|---|---|---|---|
| | | Class + ve control | | | | | Class − ve control |
| | | (HMPA) | | | | (DEMPA) | |
| Experiment | | (I) | (VI) | (VII) | (IX) | (VIII) | (X) | (II) |
| 10 μl S–9 mix 1:9 | 1 | + | + | − | − | − | − | − |
| | 2 | + | + | − | − | − | − | − |
| 50 μl S–9 mix 1:1 | 3 | + | + | + | + | + | + | − |
| | 4 | + | + | + | + | + | + | − |

cribed earlier contribute to a more accurate definition of this putative link.

It would be anticipated that the S-9 mix would be capable of oxidizing the phosphorous atom of hexamethylphosphorous amide (VI) thereby generating HMPA (I) *in vitro*. Assuming that the oxidative capacity of the S-9 mix approximates to that *in vivo*, the positive assay response produced by VI probably indicates that the presence or absence of the P=O oxygen group in a derivative of HMPA is not of major importance when assessing that compound's potential for causing cancer.

Likewise, we anticipated that the S-9 mix would convert the thio (=S) group of hexamethylphorothioic triamide (VII) into an oxo (=O) group, thereby yielding HMPA *in vitro*, and that this compound would thereby give a positive assay response. The fact that this was realized only when using the modified S-9 mix may be due to inactivation of the standard S-9 mix by sulphur radicals generated during the thione hydrolysis process (De Matteis, 1977). The modified activation system had a greater quantity of S-9 fraction per incubation tube (5·8 mg/ml as opposed to 0·245 mg/ml of total test medium) which may have allowed any destructive effects produced on the standard activation system to be absorbed, leaving a competent metabolic system capable of activating the derived HMPA. Therefore, the positive effects observed for HMPA (I), its deoxy-derivative (VI) and its thio-derivative (VII) indicate that the groups P=O, P and P=S should be regarded as equivalent (in particular to P=O) when new derivatives of HMPA are to be selected as candidates for *in vitro* evaluation.

The three alicyclic ring analogues of HMPA, compounds IX, VIII and X, were each negative when tested using the standard S-9 mix, and each was relatively nontoxic to the cells. However, when the modified S-9 mix was employed, cell toxicity increased dramatically and positive transformation effects were observed in each case. A similar effect was observed following modifications made to the chemical structure of a series of thiophene derivatives (Ashby *et al.*, 1978*b*). In that case the overall metabolism of the compounds was influenced by chemical features, whilst in the present experiments similar changes have arisen following changes made to the S-9 mix. The above 3 phosphoramide derivatives have, therefore, been defined as both reproducibly positive and reproducibly negative dependent upon the conditions of *in vitro* activation. In both circumstances the appropriate chemical class control substances were correctly identified. Were either of these controls to have changed their test response with the change in S-9 mix, serious doubts about the likely *in vivo* significance of the above three reversible positive results could have been raised. As it has been demonstrated that the change made to the cell clone (altered frequency of spontaneous transformation) could not have produced these changed responses, the change in S-9 mix must have been responsible.

A possible explanation for these effects is that one or more of the critical steps in the activation of these 3 compounds is more sensitive to the constitution of the activation system, or to the physicochemical environment of activation, than are the corresponding steps in the activation of HMPA, although even in that case the transformation frequency is marginally increased when using the modified S-9 mix (Fig. 2d). It could, therefore, be inferred that whilst these 3 compounds have been defined as potential carcinogens, the range of metabolic circumstances under which this potential will be expressed may be more limited than in the case of HMPA. If it could be shown that either one of the activation systems used was more relevant than the other to the situation *in vivo*, then firm predictions of the likely carcinogenic potential of these compounds could be made. This is not possible, in fact it is likely that both of these separate metabolic conditions, together with many others, will occur *in vivo*, if only transiently or locally in some tissues. The use of appropriate chemical class controls, therefore, contributed to an interpretation of the experimental data and may have helped to define some limiting aspects of the potential carcinogenicity of compounds IX, VIII and X.

It has already been demonstrated that changes made to the quantity of S-9 mix added to the incubation medium of an *in vitro* assay, or changes made to the S-9 fraction: co-factor ratio can modulate the *in vitro* response given by a chemical (Ames *et al.*, 1973; 1975). The present example represents the extreme case of passing from inactivity to activity *in vitro* following such changes. Whilst it could, therefore, be argued that all *in vitro* evaluations of new chemicals should be carried out using a wide range of S-9 mixes, this may be unnecessarily extreme. Rather, it may be necessary only to evaluate compounds using a small, but widely separated range of S-9 mixes in cases where an initially derived negative response is not compatible with what is already known about, or expected from, an individual chemical or class of chemicals. We have discussed elsewhere the wider implications raised by such changes to an observed *in vitro* response induced by changes made to the S-9 mix (Ashby *et al.*, 1978a; Ashby & Styles 1978*a*, *b*).

It therefore seems likely that a family of specifically substituted phosphoramides, rather than only HMPA itself, may have carcinogenic potential. Further, a unifying thread may connect this new class of potential carcinogens with the well established class of nitrosamine carcinogens. As a result it is suggested that the structural criteria established for the latter class of carcinogens may be of help in delineating the most likely potential carcinogens within the phosphoramide class.

Hexamethylphosphoramide is an extremely potent carcinogen for rats, exposure to $0{\cdot}02/10^6$ in air producing positive effects. It is, therefore, tempting to assume that any new carcinogens of this class will also be abnormally potent, but this idea should be approached with caution. The tumours produced by HMPA originated in the nasal region, during an inhalation study, and this may represent preferrential access and concentration of this unusual solvent in the membranes surrounding the convoluted turbinate bones of rats. Not only is this bone structure unrepresentative of man, but any solid derivatives of HMPA (such as VIII and IX) would be evaluated for carcinogenicity *via* the oral route, and would, therefore, be unlikely to present this problem. These compounds might well be carcinogenic, but they may possess a much lower potency than HMPA and a different target organ specificity.

There remains one apparent inconsistency with this chemical class analogy; that is, that after alpha-hydroxylation and formaldehyde release, dimethylnitrosamine (III) generates methyldiazonium hydroxide (XII), an active alkylating species (and one which is the assumed effective mutagen and carcinogenic initiator). After a similar sequence of biochemical changes had taken place with HMPA, pentamethylphosphoramide (XIII) would be formed, a compound not expected to possess alkylating properties. This could indicate that the reaction mechanism proposed for the activation of nitrosamine carcinogens (Druckrey, 1975) is only one of several possible mechanisms, another of which might also be consistent with the activation of HMPA as a mutagen and carcinogen. Consideration of these possible mechanisms will be discussed elsewhere.

The authors wish to thank Mr N. Pritchard, Mrs S. Rae and Mrs. B. Pell for technical assistance.

REFERENCES

AMES, B. N., DURSTON, W. E., YAMASAKI, E. & LEE, F. D. (1973) Carcinogens are mutagens: A simple test system combining liver homogenates for activation and bacteria for detection. *Proc. Natl. Acad. Sci. U.S.A.*, **70**, 2281.

AMES, B. N., McCANN, J. & YAMASAKI, E. (1975) Methods for detecting carcinogens and mutagens with the salmonella/mammalian-microsome mutagenicity test. *Mutat. Res.*, **31**, 347.

ASHBY, J. & PURCHASE, I. F. H. (1977) The selection of appropriate chemical class controls for use with short-term tests for potential carcinogenicity. *Ann. Occup. Hyg.*, **20**, 297.

ASHBY, J. & STYLES, J. A. (1978*a*) Does carcinogenic potency correlate with mutagenic potency in the Ames assay. *Nature*, **271**, 452; **274**, 19 (subsequent replies).

ASHBY, J. & STYLES, J. A. (1978*b*) Comutagenicity, competitive enzyme substrates and *in vitro* carcinogenicity assays. *Mutat. Res.*, **54**, 105.

ASHBY, J., STYLES, J. A. & ANDERSON, D. (1977) Selection of an *in vitro* carcinogenicity test for derivatives of the carcinogen hexamethylphosphoramide. *Br. J. Cancer*, **36**, 564.

ASHBY, J., STYLES, J. A., ANDERSON, D. & PATON, D. (1978*b*) Thiophene analogues of the carcinogens benzidine and 4-aminobiphenyl; evaluation *in vitro*. *Br. J. Cancer*, **38**, 521

ASHBY, J., STYLES, J. A. & PATON, D. (1978*a*) *In vitro* evaluation of some derivatives of the carcinogen butter yellow: implications for environmental screening, *Br. J. Cancer*, **38**, 51.

AUDRIETH, L. F. & TOY, A. D. F. (1942) The aquo ammono phosphoric acids III. The N-substituted derivatives of phosphoryl and thiophosphoryl triamides as hydrogen binding agents, *J. Am. Chem. Soc.*, **64**, 1553.

BURGADA, R. (1963) Contribution a l'étude des amides organiques de l'acide phosphoreux, *Ann. Chimie*, **8**, 347.

DE MATTEIS, F. (1977) Hepatotoxicity of carbon disulphide and of other sulphur containing chemicals: possible significance of their metabolism by oxidative desulphuration. In *Biological Reactive Intermediates, Formation, Toxicity and Inactivation.* Eds Jollow, D. J., Kocsis, J. J., Snyder, R. & Vaimo, H. New York and London: Plenum Press.

DRUCKREY, H. (1975) Chemical carcinogenesis of N-nitroso compounds. *Gann Monog. Cancer Res.*, **17**, 107.

KEAT, R. & SHAW, R. A. (1968) Phosphorus–nitrogen compounds part XXVI. The influence of benzene on the $^{1}H$ NMR Spectra of some mononuclear phosphorus (V) amides and dimethylaminocyclotriphosphazatrienes. *J. Chem. Soc.*, 703.

LEE, K. P., TROCHIMOWICZ, H. J. & SARVER, J. W. (1978) Induction of nasal tumours in rats exposed to hexamethylphosphoramide (HMPA). *Lab. Invest.*, **36**, 344.

SAUNDERS, B. C., STACEY, G. J., WILD, F. & WILDING, I. G. E. (1948) Esters containing phosphorus Part V. Esters of substituted phosphoric and phosphorous acids. *J. Chem. Soc.*, 699.

STYLES, J. A. (1977) A method of detecting carcinogenic organic chemicals using mammalian cells in culture. *Br. J. Cancer*, **36**, 558.

VETTER, H. J. & NÖTH, H. (1963) Dialkylaminophosphane IV, Additionen und Substitutionen am tris(dimethylamino)phosphan, $P(NMe_2)_3$. *Ber*, **96**, 1308.

ZAPP, J. A. (1975) Inhalation toxicity of hexamethylphosphoramide. *Am. Ind. Hyg. Ass. J.*, **36**, 916.